# Sleep quality and cognitive functioning among Chinese older adults living in the US

**DOI:** 10.1093/geroni/igaf122.3235

**Published:** 2025-12-31

**Authors:** Guoping Jin, Fengyan Tang

**Affiliations:** University of Pittsburgh, Pittsburgh, Pennsylvania, United States; University of Pittsburgh, Pittsburgh, Pennsylvania, United States

## Abstract

Racial and ethnic disparities in sleep quality and cognitive health are increasingly recognized, yet little is understood about their associations among Chinese older adults living in the U.S. This study aims to examine the relationships between sleep health and cognitive functioning in this population. This observational study utilized a two-wave panel design as part of the Population Study of Chinese Elderly in Chicago, including 2,228 participants aged 65 years or older who self-identified as Chinese. Participants completed interviews at two time points. Mixed-effects regression models were used to assess the effects of sleep parameters on baseline cognitive functioning and cognitive change over time. Participants had an average age of 77.42 years (±7.57) at baseline, with about 39% reporting fairly bad or very bad sleep quality. Poorer overall sleep quality (B = -0.01, SE = 0.01, p<.01), and more insomnia symptoms (B = -0.01, SE = 0.00, p<.001) were associated with lower baseline global cognition. However, these associations diminished over time (sleep quality: B = 0.01, SE = 0.00, p<.05; insomnia: B = 0.00, SE = 0.00, p<.05). Among sleep quality subdomains, all except sleep efficiency had significantly negative relationships with baseline global cognition. The associations between sleep parameters and the four cognitive domains were less consistent. The findings highlight cross-sectional negative relationships between self-reported sleep parameters and cognition, showing distinct associations between various aspects of sleep quality and cognitive domains. Targeted interventions to improve sleep quality may have the potential to enhance cognitive health outcomes.

